# Acute phase of aortic dissection: a pilot study on CD40L, MPO, and MMP-1, -2, 9 and TIMP-1 circulating levels in elderly patients

**DOI:** 10.1186/s12979-016-0063-2

**Published:** 2016-03-22

**Authors:** E. Vianello, E. Dozio, R. Rigolini, M. M. Marrocco-Trischitta, L. Tacchini, S. Trimarchi, M. M. Corsi Romanelli

**Affiliations:** Department of Biomedical Sciences for Health, Università degli Studi di Milano, Via Luigi Mangiagalli 31, 20133 Milan, Italy; Laboratory Medicine Operative Unit-1, Clinical Pathology, I.R.C.C.S. Policlinico, San Donato Milanese Milan, Italy; Thoracic Aortic Research Center, I.R.C.C.S. Policlinico San Donato, San Donato Milanese Milan, Italy

**Keywords:** Acute aortic dissection (AAD), CD40 ligand (CD40L), Matrix metalloproteinases (MMPs), Metallopeptidase tissue inhibitor 1 (TIMP-1), Myeloperoxidase (MPO)

## Abstract

**Background:**

Acute aortic dissection (AAD) is an event which may be rapidly fatal without early diagnosis and treatment. Aging is one of the main risk factors that could leading to AAD. To date, no specific biomarkers are available to increase the speed of diagnosis. CD40 ligand (CD40L), myeloperoxidase (MPO), matrix metalloproteinase (MMP)-1, -2, -9 and metallopeptidase tissue inhibitor 1 (TIMP-1) are biologically related molecules which integrate inflammation, tissue injury and remodeling, all events associated to AAD.

Our is a pilot study to evaluate whether circulating levels of these molecules may be used as potential biomarkers in timely diagnosis of AAD.

**Results:**

Within 24 h of symptom onset, circulating CD40L, MPO, MMP-1,-2,-9 and TIMP-1 were quantified by enzyme-linked immunosorbent assays in 22 patients (40–86 years of age) with AAD of ascending aorta (type A according to Stanford classification) and 11 patients with AAD of descending aorta (type B). 30 healthy individuals age matched were used as control group compared to controls, both type A and B AAD patients had higher CD40L (*p* < 0.001) and MPO (*p* < 0.01) levels. MMP-1 was higher in the overall AAD group (*p* < 0.01). After Stanford classification, type A group had increased level compared to both control and type B (*p* < 0.01 and *p* < 0.05, respectively). TIMP-1 was higher in both A and B groups compared to controls (*p* < 0.001). No differences were observed in MMP-2 and MMP-9 levels.

**Conclusions:**

The simultaneous evaluation of CD40L, MPO and MMP-1 and TIMP-1, which may contribute to structural changes in aortic tissue in AAD patients, seems to be a novel promising diagnostic panel.

## Background

Acute aortic dissection (AAD) is a clinical condition caused by a circumferential or, less frequently, transverse tear of the intima. The initiating event is either a primary intimal tear with secondary dissection into the media or a medial hemorrhage that dissects into and disrupts the intima. The etiology of AAD is imputed to either genetic disorders affecting connective tissue, such as Marfan syndrome, or as a consequence of primary disorders, generally called non-Marfan AAD [1, 2]. Aging is the main of all risk factors leading to AAD including atherosclerosis or hypertension [3, 4]. Particularly it has been known that patients with AAD undergoing to coronary by pass surgery with 40–86 years of age range had a really dramatically decrease in the number of interlaminar fibers and an increase of laminar distance with aging [5, 6]. This pathway could result in a dilatation of the aorta and incurs in AAD [7].

In particular the pathophysiology of AAD was characterized by degeneration of the aortic fascia, smooth muscle cell (SMC) loss, inflammation, fragmentation and depletion of elastic fibers [8, 9] that are key events in AAD which may lead to recruitment of local T-cells and macrophages as a primary response to endothelial damage [9, 10]. These assumptions were well supported in previous study focused on the identification of different mechanisms that can contribute to the up-regulation of genes involved in the endothelial turnover and also it is well reported that a differential genetic profiles can be related to aortic dissection development [11–13]. In fact genomic alterations of enzymes involved into endothelium turnover, as the metalloproteinases (MMPs), and polymorphisms of their genes, were directly associated to aortic degeneration due to the fact MMPs are deputed to maintain elastin and collagen in aorta composition during endothelial damage [14, 15].

Furthermore AAD may be rapidly fatal without early diagnosis and treatment [16–18]. Among different mediators that can be induced during endothelial turnover after AAD lesion, CD40 ligand (CD40L), myeloperoxidase (MPO), matrix metalloproteinases (MMPs), and metallopeptidase tissue inhibitor 1 (TIMP-1) are biologically related molecules which integrate inflammation, activation of different immune cells, tissue injury and remodeling, all events associated to ADD. In fact, CD40L on the surface of activated T-cells, binds the CD40 receptor of macrophages. These cells are then induced to release MMPs, in particular MMP-1, which promote the initial phase of vascular extracellular matrix degradation [19, 20]. In normal conditions, this mechanism is naturally regulated by TIMP-1 which can balance increased MMPs activities [21]. Amplification of inflammation is also sustained by recruitment of other immune cells. In particular, neutrophils are recruited in response to endothelial damage and the increased production of myeloperoxidase (MPO) is a marker of their activation [22, 23].

To date, AAD diagnosis is based on symptoms, history and physical examination, electrocardiography, chest X-rays and imaging studies. No specific biomarkers are available to increase the speed of diagnosis.

To our knowledge alteration of molecules associated to endothelial damage during the early stage of AAD have not been fully explored and for this reason our study should be considered as a pilot study aimed to evaluate whether the simultaneous evaluation of circulating levels of CD40L, MPO and MMP-1,-2,-9 and TIMP-1 could represent a novel powerful diagnostic tool in timely diagnosis of AAD.

## Methods

### Patients

We enrolled at I.R.C.C.S. Policlinico San Donato of Milan 32 male patients (40–86 years of age, mean 62.2 ± 18.6 years) with only non-Marfan AAD to exclude any possible genetic confounding factors, diagnosed within 24 h of symptom onset and 30 healthy age-matched individuals as a control group. AAD patients were then classified according to Stanford classification in two groups: type A (*n* = 22) including patients in which the dissection involves the ascending aorta (proximal dissection) and type B (*n* = 11) including patients in which the dissection is limited to the descending aorta (distal dissection). Hospitalized type A AAD patients underwent to vascular surgery, presented first blood systolic pressure with average value of 126 mmHg and a diastolic pressure of 70 mmHg. All type A patients had history of hypertension and only 2 patients had history of atherosclerosis. Type A patients were treated with beta and calcium channel blockers and ACE inhibitors. Hospitalized type B AAD patients, underwent to endovascular management, had a first blood systolic pressure with average value of 206 mmHg and a diastolic pressure of 120 mmHg with history of atherosclerosis, treated with nitroprusside drug and calcium channel blockers. Both type A and B patients have not of prior aortic dissection and don’t have prior mitral, bicuspid, cuspid and aortic valve diseases. All patients enrolled in this study weren’t smokers and don’t have abused of cocaine drug.

The study protocol was approved by local Ethics Committee (ASL Milano 2) and patients gave their written informed consent, conducted in accordance with the Declaration of Helsinki, as revised in 2013.

### Experimental

Plasma sample were collected immediately after ospedalization, before the surgery and separated after centrifugation at 1000 g for 15 min and were stored at −20 °C until analysis. The quantification of CD40L, MPO, MMP-1,-2,-9 and TIMP-1 and were measured by enzyme-linked immunosorbent assays (ELISA) according to the manufacturer’s directions (Quantakine Immunoassay, R&D System, Minneapolis, Minnesota).

### Statistical analysis

Data were expressed as mean ± standard deviation (SD) and analyzed by GraphPad Prism 5.0 biochemical statistical package (GraphPad Software, Inc., San Diego, CA). The normality of data distribution was assessed by the Kolmogrov-Smirnoff test. Comparison between groups was performed using Student’s two-tailed unpaired T-test or Mann-Whitney U-test, as appropriate. A *p* value < 0.05 was considered statistically significant.

## Results

**Fig. 1:**
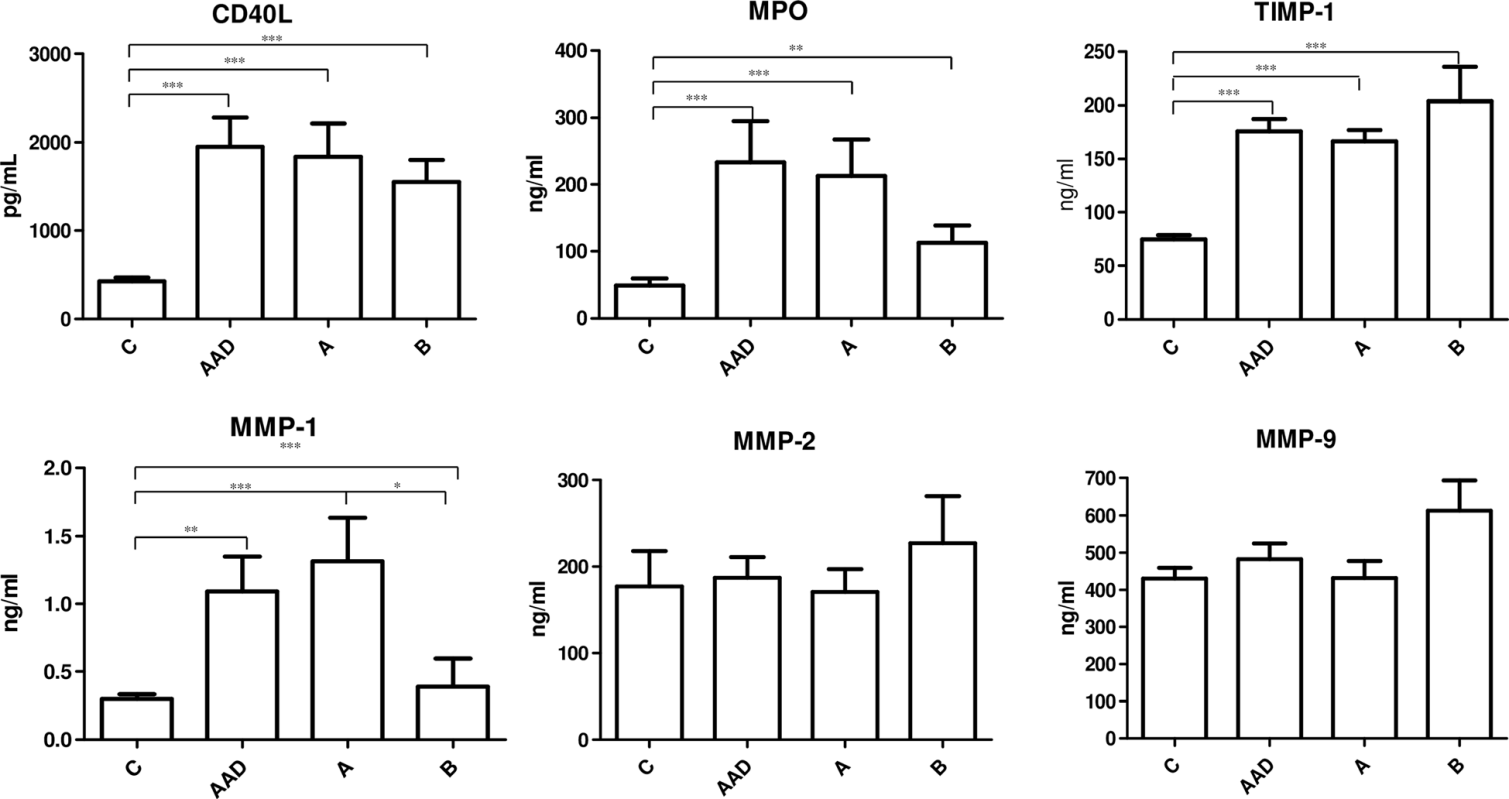
Circulating levels of molecules involved into endothelium remodeling in aortic dissection and controls patients. AAD patients have higher level of CD40L, MPO, TIMP-1, and MMP-1 than controls (*p* < 0.05). The same trend was also highlighted subdividing AAD patients in type A and B according to Stanford classification (*p* < 0.05). No statistically significance was observed in MMP-2 and MMP-9 circulating levels between AAD and control patients. Between A and B subgroups of ADD patients any statistically significance was observed (*p* = 0.05)

Compared to controls, both type A and B AAD patients had higher CD40L (*p* < 0.001) and MPO (*p* < 0.01) levels (*p* < 0.001 for both) and MPO (*p* < 0.01 for both) (Fig. 1).

MMP-1 was higher in the overall AAD group (*p* < 0.01). After Stanford classification, type A group had increased level compared both to controls and type B (*p* < 0.01 and *p* < 0.05, respectively). Controls and type B patients were almost the same (Fig. 1).

TIMP-1 was higher in the overall AAD group, as well as in both type A and B, compared to controls (*p* < 0.001 for all).

Any difference was found in MMP-2 and MMP-9 levels nor between controls and AAD patients, nor after classification (*p* > 0.05 for all).

## Discussion

Our findings indicate that during the acute phase of aorta dissection within 24 h, compensatory mechanisms to overcome the endothelial damage result in increase of the molecules involved in tissue remodeling as well as the mediators of immune response. Our result shown that CD40L levels were markedly higher in AAD patients – aimed at intensifying macrophage and T-cell recruitment – as proved also by the higher MPO levels in the same group. This confirm that not only in late stage but also in the early phase of AAD, the endothelium instability is mediated first by pro-inflammatory cytokines and chemoattractants T linfocytes. Secondly, the local induced macrophages are stimulated to release MMPs aimed to disintegrated extra-cellular products from endothelial rupture.

Another important point is that the endothelial fragmentation of elastin and collagen occurs not only in Marfan syndrome but also in spontaneous AAD and MMP and they play a pivotal role in elastin and collagen digestion and consequently into endothelium turnover and repair as previously described [19, 24].

Among MMP, it is reported that MMP-1 is the principal enzyme involved to endothelium remodeling due to its ability to degrade type I and III collagens that are the major collagens present in vascular wall. This assumption is well demonstrated by our results which highlighted in AAD patients higher levels of MMP-1 compared to controls.

Contrarily, any statistical differences were observed in both MMP-2 and MMP-9 levels as expected. This may be explained by two principal reasons: first MMP work together but the first enzyme activated by vascular wall damage is MMP-1 followed by the further cleavage of MMP-2 and then of the other MMP [19, 24].

Secondly, in addition to the activity of TIMP-1, the natural inhibitor of MMP and in particular of MMP-9, it has demonstrated that the levels of MMP-9 aimed to degrade elastin fragments, can dramatically decrease due to the massive MMP-1 which can reduce elastin availability [19]. Therefore we can suggest that MMP-1, well recognized to be the principal mediator of vascular remodeling in overt AAD, can be also an important marker of AAD in the early stage since its levels change yet within 24 h of signs and symptoms of this disorder.

## Conclusions

Our study is one of the few studies focused on the early stage of AAD in particular within the 24 h from signs and symptoms of this disorder. Although previous studies demonstrated the simultaneous activation and increased levels of all MMPs and their inhibitor during aortic dissection, their observations were mainly done after the overt stage of the dissection, specifically over the 24 h from signs and symptoms of the disorder and mainly after surgery.

Contrarily our study was done during the early stage of the dissection in which MMP-1 is the first MMP that increase its levels in the early phase of the lesion in occurrence of CD40L, MPO and TIMP-1 increases.

On the light of this, we finally suggest the simultaneous evaluation of CD40L, MPO and MMP-1 and TIMP-1 circulating levels as promising diagnostic tool for clinical assessment in the early phase of AAD although additional experiments are need to confirm our outcomes.

## Abbreviations

AAD: acute aortic dissection
CD40L: CD40 ligand
MPO: myeloperoxidase
MMP: matrix metalloproteinase
TIMP-1: metallopeptidase tissue inhibitor 1
